# Assessment of Risk Factors, Prehospital Measures and Clinical Needs of Patients Admitted With Snake Envenomation at a Rural Hospital in Trinidad and Tobago

**DOI:** 10.7759/cureus.29616

**Published:** 2022-09-26

**Authors:** Darren Dookeeram, Satesh Bidaisee, Caitlyn Hatcher, Nicole Nguyen, Sandeep Maharaj

**Affiliations:** 1 Emergency, Sangre Grande Regional Hospital, Sangre Grande, TTO; 2 Public Health, St. George's University, St. George's, GRD; 3 Pharmacy, University of the West Indies, St. Augustine, TTO

**Keywords:** occupational hazards, farmers, snake-bite, pre-hospital, envenomation

## Abstract

Objective: This study assessed the risk factors for sustaining a snake bite, the prehospital measures employed, and the clinical needs of patients admitted with confirmed envenomation.

Methodology: Data was collected at a single center, a rural secondary care hospital in Trinidad and Tobago. A cross-sectional method was used that enrolled all consenting patients requiring admission following clinical confirmation of snake envenomation during the period 2017-2019. Data collection involved a review of the patient record from the emergency room and hospital admission to establish the clinical need during the time of admission which was defined as the administration of medication, need for surgery, and critical care intervention. Data collection also involved a patient interview to establish demographics, prehospital measures employed, and assessment of the risk factors associated with sustaining the snake bite.

Results: 29 patients were admitted for snakebite envenomation during 2017-2019 and all patients consented to enrolment. Of these patients, 22 were male and most commonly were within the age range of 18-40 years old. 34.5% of patients were farmers and 68.9% of patients identified being bitten by the Mapepire Balsain snake. 65.5% of patients reported being unaware of the risk of snake bites and 82.8% were not wearing boots, with the lower limb being the most common bite site among 55.2%. 41.4% of bites were sustained during work-related activity while 34.5% of bites were sustained during recreational activity. Prehospital measures were employed by 18 of the 29 patients with the most common types being irrigation (10.3%), cutting (6.9%), tourniquets (44.8%), pressure immobilization (6.9%), topical applications (3.4%), and ingestion of a substance (6.9%). 34.5% received hospital care within 1 hour of the bite while 55.2% arrived at the hospital between 1 and 4 hours of being bitten. The clinical challenges of these patients included local reactions (82.8%), coagulopathy (72.4%), compartment syndrome (17.2%), cellulitis (3.4%), and dislocated shoulder (3.4%). The clinical needs of these patients included vitamin K (13.8%), antibiotics (93.1%), tetanus shots (17.2%), analgesia (6.8%), and anti-venom (82.7%). 10.3% of patients required debridement and 3.4% required a fasciotomy. The average stay in the hospital was 3.8 days. There were no documents of deaths or need for critical care.

Conclusion: Persons are most likely to be envenomated by the M. Balsain in Trinidad. These patients are commonly males ranging anywhere from 18 to 40 years presenting local reactions and coagulopathy needing admittance to the hospital. While the majority of patients requiring admission performed some type of prehospital measure, very few did so with pressure immobilization. Furthermore, the majority of patients had a prolonged time before presenting to the hospital; this is a potential area for improvement in the health system through education and sensitization. There was a significant utilization of resources on these patients when taking into consideration their clinical needs, medication, and hospital stay; primary prevention should be a focus through the education of groups who are at higher risk for a venomous snake encounter.

## Introduction

According to the World Health Organization (WHO), snakebite remains a relevant and important public health problem in rural areas of tropical and subtropical countries.; this is underscored by its 2017 inclusion in the top 20 neglected tropical diseases [1]. The WHO's most recent reports continue to estimate the annual incidence of snakebite as high as 5.4 million with up to 2.7 million developing critical illness and as many as 138,000 deaths but clarifies that these numbers may be a gross underestimation because of underreporting of incidence and mortality in rural communities [2]. Other studies estimating the burden of morbidity and mortality of snakebites conclude that deaths from snakebites may actually range between 1.2 and 5.5 million annually [3]. Since snakebite is more likely in rural communities, it is thought that morbidity and mortality are underreported, because of poor access to health services, scarcity of antivenom, and a propensity of victims to seek care from traditional healers [4]. Four major families of snakes are considered dangerously venomous and of medical importance. These include Elapidae (cobras, kraits, mambas, coral snakes, Australasian snakes, sea snakes), Viperidae (old world vipers and adders, American rattlesnakes, moccasins, lance-headed vipers, Asian pit vipers), Atractaspidinae (burrowing asps) and Colubridae (arboreal back-fanged snakes) [5].

A 2004 publication by Sammy et al. found the number of snakebites at the Sangre Grande Hospital in Trinidad and Tobago, to be 29 over a one-year period [6]; making it a relatively common disease in a rural community (population 15,968) of a Small Island Developing State (population 1,531,266) [7]. The population described in that study is the same as was assessed in this paper, however, no other published epidemiological data is available to date. This persisting paucity of data prompted the formation of the Poison Information Center (PIC) in 2006 as an arm of the Ministry of Health in conjunction with the University of the West Indies that allows more standardized data collection of incidences, use of antivenin, and all-cause morbidity and mortality [8]. According to the Nature Center of Trinidad and Tobago, two snakes on the islands are considered venomous which are the coral snakes and mapepire (pit viper) species with the majority of envenomation resulting from Bothrops antrox (Fer-de-Lance) pit viper species [9]. In addition to information regarding the identification of potentially poisonous species, the Nature Center makes information available to the public on tips to avoid being bitten and key prehospital measures in case of a bite; these reflect the WHO guidance as described below [9].

The WHO management guideline of envenomation requires immobilization of the bitten limb, reassurance, and prompt hospital assessment for the patient; the guideline strongly discourages the use of traditional remedies since they have been found to cause more harm [1]. Despite the existence of this evidence, it is still commonplace for rural communities to institute such treatments which may ultimately cause harm to the patient [10]. It is therefore imperative that persons who deliver first aid in rural communities with persons at risk be educated about the need for urgent hospital care and avoidance of unnecessary interventions [11]. Additionally, there is evidence that at-risk populations should be educated about measures that could decrease the likelihood of exposure to envenomation such as protective clothing [12].

The purpose of this study was to establish the updated prevalence of envenomation at the Sangre Grande Hospital requiring admission, the risk behavior associated with the snake encounter, the frequency of prehospital measures, the morbidity, and mortality, and the impact of the snake encounter on risk behavior.

## Materials and methods

This study utilized a cross-sectional analysis of adults admitted to the Sangre Grande Hospital in Trinidad and Tobago. The Sangre Grande Hospital is the flagstaff secondary care institution of the Eastern Regional Health Authority and serves a largely rural population of the country with significant agricultural and eco-tourism interests. Beyond this, it represents the hospital serving approximately 120 000 people and one-third of the land mass of Trinidad which is the largest geographical area of all regional health authorities in Trinidad and Tobago. The hospital houses a fully functional emergency department and multiple specialties including internal medicine, general surgery, and critical care [13]. The geographical area that the ERHA serves corresponds to the more agricultural parts of the country and as such, sees the majority of snakebite victims in the country. As such the PIC has been strategically embedded within this region as a coordinating body for rapid information dissemination and treatment of such problems. Ethical approval was obtained from the Eastern Regional Health Ethics Committee and the Sangre Grande Hospital Administrative office.

Records review

The medical records from the emergency department and hospital admission were reviewed to collect data on the patient’s vitals, treatments, surgical interventions, and disposition. The clinical presentation and location of the snakebite were documented, and it was noted if the patient presented with a local reaction, compartment syndrome, shock, coagulopathy, or necrosis. Treatment data were also collected, including what medications were given, such as steroids, antibiotics, and antivenoms. It was also noted if the patient received any surgical interventions, such as fasciotomy, debridement, or amputation of a limb. Lastly, data was collected on the patient’s disposition from the hospital, including the total time admitted in the ward and when the patient was discharged from the hospital.

Patient interview

A patient interview was conducted to collect data regarding demographics, risk assessment, and prehospital measures taken. The demographic information collected included age, gender, occupation, and geographical region, as well as what activity was being performed at the time of the bite. A risk assessment questionnaire was also given to assessing the patient’s awareness of the possibility of being bitten by a snake at the time of the encounter and if any protective measures were taken. Additional information was collected regarding the incident surrounding the snakebite, including what type of snake caused the bite, what time of day the bite occurred, and what circumstance was associated with the activity surrounding the bite. The circumstances listed include whether the activity was occupational, recreational, in-house, pet, or other.

Lastly, data were collected on any prehospital measures taken immediately following a snakebite. The prehospital measures taken included irrigation, suction, or cutting of the wound; application of a poultice, tourniquet, or topical substance; ingestion of a substance; or pressure immobilization. This also included the time it took the patient to reach a hospital or healthcare provider following envenomation.

Data security

Patient confidentiality was maintained by keeping all data collected anonymously. No markers bearing patients’ personal information were used after the data had been obtained from the medical records. Only the primary investigator will have access to the patient log from the PIC and the patient records and no unique identities will be attached to questionnaires.

Data analysis

Information gathered via collection sheets was entered into a database corresponding to the questionnaires. Subsequent data analysis will be performed using SPSS version 7.0 (IBM Corp., Armonk, NY) to check for the outcomes listed in the primary and secondary questions.

## Results

A total of 29 patients were admitted during the time under consideration in this study. The demographic breakdown is illustrated in Table 1.

**Table 1:** Demographic features

Variable	n (%)
Gender	
Male	22 (75.8)
Female	6 (20.7)
Age	
18-40	13 (44.8)
41-65	12 (41.3)
>65	4 (13.7)
Occupation	
Farmer	10 (34.5)
Non-farmer	19 (65.5)
Snake species	
M. Balsain	20 (68.9)
Not identified	5 (17.3)
Coral	2 (6.8)
M. Zanana	2 (6.8)

The majority of patients hospitalized with envenomation during the period of the study were males (75.8%). The age distribution was fairly equal between the 18-40 and 41-65 age groups, each with just over 40% of the sample size of the group. There were fewer persons over the age of 13.7%. Regarding occupation, ten individuals were farmers (34.5%) and the remaining nineteen were non-farmers (65.5%). Some of the non-farmer occupations listed included truck drivers and laborers who practically have similar exposure profiles as those who are full-time farmers. The majority of bites were from M. Balsain (68.9%) with 20 total cases, followed by unknown/other (17.3%) with five total cases, then coral and M. Zanana equally (6.8%) with two cases each. Three of the four “other” snake types came from the Mapepire species. The geographical location of these cases was spread out throughout Trinidad, with the most bites occurring in the regions of Rio Claro (13.8%) and Valencia (13.8%) with four cases each.

As demonstrated in Table 2, the month of occurrence peaked in September, which had the highest incidence of six cases (20.6%), followed by five cases in October (17.2%). The lowest number of cases occurred in January and February with only one case occurring during each month (3.4%).

**Table 2:** Risk factors for snake bite

Variable	N (%)
Time of day	
6am – 12 noon	12 (41.3)
12 noon – 6pm	12 (41.3)
6pm – 6am	5 (17.2)
Activity	
Occupational	12 (41.3)
Recreational	10 (34.5)
Within house	3 (10.3)
Other	3 (10.3)
Hunting	1 (3.4)
Pet	0 (0)

The time of day varied, with twelve cases occurring between 6am-12pm (41.3%), twelve cases occurring between 12pm-6pm (41.3%), and five cases between 6pm-6am (17.2%). Regarding the circumstances surrounding the bite, the most common associated activity was listed as “occupational” (41.4%), followed by “recreational” activities (34.5%) and “in-house” activities (10.3%). Three individuals listed “other” activities as the circumstance (10.3%), with two of these cases specifying that they were walking when they were a bit. One individual indicated that they were hunting at the time of the bite (3.4%). There were no cases associated with “pet” activities, meaning no bite was caused by a pet snake.

Table 3 demonstrates the use of protective measures and illustrates that the majority of people who were bitten were not wearing boots (82.7%) compared to those who were wearing boots (17.2%). Similarly, the majority of people who were bitten were not wearing gloves (96.5%) compared to one person who was wearing gloves (3.4%). While there was an increase in the number of persons who stated that they would wear boots and gloves when returning to their normal activity, the increase was to about 50%, however, those who would warn others about the risk of snakebites increased to about 75%.

**Table 3:** Personal protective equipment and awareness

Variable	Yes, n (%)	No, n (%)	Unsure, n (%)
Were you wearing gloves?	1 (3.4)	28 (96.5)	
Were you wearing boots?	5 (17.2)	24 (82.8)	
Were you aware of the risk of a snakebite?	8 (27.6)	19 (65.5)	2 (6.9)
Has anyone ever warned you about the risk of snakebite?	8 (27.6)	20 (68.9)	
Were other members of your family with you at the time?	13 (44.8)	16 (55.1)	
When you return to work, will you wear boots?	13 (44.8)	15 (51.7)	1 (3.4)
Will you warn others about the danger of snakebite?	21 (72.4)	5 (17.2)	3 (10.3)
Will you warn others about wearing gloves and boots?	22 (75.9)	4 (13.8)	3 (10.3)

As demonstrated in Table 4, there was a wide range of prehospital measures that were performed, three (10.3) persons sought access to care from traditional healers before accessing the healthcare system. Twenty-two patients partook in prehospital measures, with tourniquet application being the most common (44.8%), followed by irrigation of the wound (10.3%). Cutting of the wound (6.9%), pressure immobilization (6.9%), and ingestion of a substance (6.9%) was next, with two cases each. Those who ingested a substance included one person ingesting olive oil and another ingesting a handful of dirt. One person used topical application of a substance as a prehospital measure, which was noted to be a cigarette burn onto the bite wound. In terms of how long it took the patient to get to a healthcare provider following a snakebite, most individuals took between 1 and 4 hours to get to a hospital (55.2%). Ten individuals took less than 1 hour (34.5%), two people took 4-24 hours (6.9%), and one person took more than 24 hours to get to a hospital (3.4%).

**Table 4:** Prehospital measures employed

Variable	N (%)
Irrigation	3 (10.3)
Suction	0 (0)
Poultice	2 (6.8)
Cutting	2 (6.9)
Tourniquet	13 (44.8)
Pressure Immobilization	2 (6.9)
Topical application	1 (3.4)
Ingestion of substances	2 (6.9)

Table 5 reflects the clinical findings in the population. In terms of the site of envenomation, there was no limb that showed a significantly higher likelihood of being affected. The most common bite site was the right lower limb (31%), followed by the left upper limb (27.5%), left lower limb (24.1%), and right upper limb (17.2%). There was also a wide spectrum of presenting complaints and findings but the most commonly noted features included local reaction (82.8%), coagulopathy (72.4%), and compartment syndrome (17.2%). Additional presenting features included three patients in hypertensive crisis (systolic over 180 and/or diastolic over 120) (10.3%), seven patients presented with tachycardia (heart rate greater than 100 bpm) (24.1%), and two patients were hyperventilating (respiratory rate greater than 20 breaths per minute) (6.9%), one patient had a low temperature of 34.7°C (94.4°F) and one patient had a mildly elevated temperature of 38.2°C (100.7°F). Orthopedic complications included one patient having cellulitis (3.4%) and another having a dislocated shoulder (3.4%) that resulted from a fall after the snake bite had occurred.

**Table 5:** Site of Bite and Clinical Presentations

Variable	N (%)
Site of Bite	
Right Lower Limb	9 (31)
Left Lower Limb	7 (24.1)
Right Upper Limb	5 (17.2)
Left Upper Limb	8 (27.5)
Common Clinical Presentations	
Local reaction	24 (82.8)
Coagulopathy	21 (72.4)
Compartment syndrome	5 (17.2)

Table 6 reflects the medical and surgical clinical needs of patients sustaining envenomation during the period of the study. It should be noted that the administration of antibiotics, antihistamines, and steroids was done at the district health facilities before hospital transfers. Hospital interventions included antivenom and fresh frozen plasma (FFP) based on local guidelines. Regarding medications given, antibiotics were the most common (93.1%), followed by steroids (89.6%), antihistamine (86.2%), antivenom (82.7%), vitamin K (13.8%), and FFP (6.8%). Five patients received a tetanus vaccine (17.2%) and two patients received analgesia (6.8%). The number of antivenom vials given varied from none to 15 vials given, with the average being 8.3 vials. As for treatments given, three patients required debridement (10.3%), one patient required a fasciotomy (3.4%), and one patient required aspiration (3.4%). No patients required amputation. All patients were admitted to the ward following an evaluation from the emergency department. The number of days in the ward varied from one day to 11 days, with 3.8 days being the average amount of time spent in the ward. M. Balsain cases had the longest average hospital duration at 4.4 days, with one case lasting 11 days, and another lasting 9 days. No patients were admitted to the ICU, and no patients expired. The data are seen in Table 7.

**Table 6:** Medical and Surgical Clinical Needs

Intervention	N (%)
Medical	
Antibiotics	27 (93.1)
Steroids	26 (89.6)
Antihistamine	25 (86.2)
Antivenom	24 (82.7)
Vitamin K	4 (13.8)
Fresh Frozen Plasma	2 (6.8)
Tetanus Vaccine	5 (17.2)
Anti-inflammatory	2 (6.8)
Surgical	
Debridement	3 (10.3)
Fasciotomy	1 (3.4)
Aspiration	1 (3.4)
Amputation	0 (0)

**Table 7:** Snake type vs. average hospital duration.

Snake Type	Average Duration of Hospital Stay (days)
M. Balsain	4.35
Coral	2
M. Zanana	2
Unknown	1
Average Stay	3.77
ICU stays	0

## Discussion

The literature reflects that the risk factors for sustaining a snake bite are influenced by a wide range of factors, but common themes suggest that middle-aged, male, field workers with low levels of education are at the greatest risk [14]. It is noted by Trinidadian ophiologist Hans Boos that it is a common practice that snakes are killed in many cultures, regardless of whether they are venomous or not. This has the potential to unnecessarily increase the interface between snakes and humans and inherently increases the risk of persons sustaining life-threatening envenomation [15]. Furthermore, it is globally recognized that underreporting of snake bites is due to a lack of access to health services, dependence on traditional healers, and lack of sensitization on the subject matter [16]. All of these factors contribute to the increased likelihood of morbidity and mortality associated with this condition.

In this study, people who were more likely to be bitten were males under the age of 65. Males made up 75.8% of the snake envenomation reported in this study. This pattern seems well described in the literature on this topic and is multifactorial and culture specific. Furthermore, 89.7% of individuals were under 65 years of age. This could be due to people older than 65 being less likely to participate in activities that would put them into contact with snakes such as farming and recreational activity [17].

Snakebite has been linked to wet seasons. This is due to the rain-washing snakes out from the higher ground to the lower ground [18]. The wet season in Trinidad usually begins sometime in May and ends in November [19]. The majority of the snake bites reported in this study occurred during that time frame of the wet season, but it should be noted that some agricultural activities and recreational activities peak during the dry season, and this can be a potential risk factor. Out of the 29 individuals who were admitted, 20 were attributed to M. Balsain's envenomation. This is most likely due to the aggressive nature of the snake, its ability to blend in with the environment, and its being active during the day. Both the coral snake and M. Zanana are nocturnal snakes. Only five snake bites occurred during the hours of 6pm and 6am. Coral snakes M. Zanana are also known to be more docile than M. Balsain [15].

Knowledge of the possibility of being bitten by a snake had very little effect on people taking precautions such as boots and gloves. Only eight people stated that they were aware that there was a possibility of being bitten by a snake. Out of those eight people, none of them were wearing gloves and one was wearing boots. Four out of the five individuals who reported wearing boots were farmers. In a detailed guideline from the government of Canada, the level of personal protective equipment (PPE) that farmers should be provided with included foot and hand protection in addition to hearing, eyes, respiratory, head, and body [20]. It should be noted in this context that the majority of farmers in Trinidad and Tobago are self-employed and unregulated. While a more detailed analysis of the use of PPE in the farming community is indicated, this study suggests that there is an urgent need for mass sensitization programs to ensure the safety of this vital, yet vulnerable, group in society.

Since knowledge of being bitten by a snake has little bearing on whether an individual takes extra safety precautions then any effort in education should focus on first aid protocols and clearing up misconceptions regarding the prehospital treatment of snake bites. The protocol set forth by the WHO states that the treatment for snake bites should include reassuring the victim to decrease anxiousness, immobilizing the patient (pressure immobilization should be used if the snake might have a neurotoxic venom), and avoiding any contact with the wound that might result in infection. Above all, it is emphasized that the patient should immediately be transported to a location that can provide definitive care [1].

The majority of individuals interviewed used tourniquets, which are contraindicated for snake bite due to the risk of causing tissue ischemia and gangrene [1]. Other measures used included cutting, irrigation, topical application, and ingestion of substances. All of these are contraindicated due to increasing the time between envenomation and transport, and the possibility of introducing infection to the wound [1]. All but three of the patients arrived at the hospital within 4 hours of envenomation. It should also be noted that the island of Trinidad is only 1,850 square miles (4,800 square km), so the distance to definitive care is not as far as other nations that are afflicted by snakebite [21]. From a health systems point of view, it is essential that farming communities be given equitable access to the required care that may be potentially indicated. It is often the case that these rural areas associated with agricultural services remain underserved since the focus remains on urban centers where disease burdens are higher because of the population density. The onus, therefore, rests with the health systems analysts to ensure that the vulnerable communities being discussed be given some mechanism to grow trust in the health system and be given direct or indirect access to service. This study showed no correlations between time from envenomation to hospital and the outcome of the patient. In fact, the only individual who took more than 24 hours to seek treatment at the hospital spent only one day on the ward. This patient also was bitten by an unknown snake but did have coagulopathy symptoms, which would lead to the conclusion that it was one of the hemotoxic snakes on the island.

Most of the symptoms that patients presented with were hematological due to most of the envenomation coming from snakes that have hemotoxic venom (M. Balsain) [9]. The two most common were local reactions and coagulopathy. Five individuals had compartment syndrome. All five of the patients with compartment syndrome used a tourniquet as part of their prehospital treatment. Seven patients required some sort of surgical intervention, none of these could be linked to their prehospital care. It is well demonstrated in the literature that the foremost prehospital measure that is recommended is immobilization to prevent the lymphatic spread of the toxin. Systemic reviews have repeatedly listed the negative effects of the use of tourniquets and, in some cases, pressure immobilization bandages that are improperly applied [11]. The practical point, in this case, is that populations that are at risk for these events should have appropriate training and sensitization. Furthermore, as already discussed, there is high utilization of local healers in rural communities. The onus therefore should be on policymakers and health administrators to form meaningful relationships with unregulated providers to ensure that potentially life and limb-saving techniques can be delivered in the communities in a timely manner.

None of the individuals in this study died due to the envenomation. All were admitted to the wards for further treatment and observation. Even though the use of the contraindicated measures did not result in death, it possibly could have resulted in serious permanent injury. In order to see if there were any detrimental effects due to the use of contraindicated treatments further study would be needed.

Limitations

This study had a small sample size. Even with this, valuable information regarding the demographics of the individuals most prone to snake bite and how they are most likely to present. The information obtained can also help focus community education regarding snake bites, which will hopefully lead to better outcomes and decreased snake bite occurrences. More studies are needed in the Caribbean and other regions regarding snake bites due to each region's unique traditions and beliefs regarding snakes and snake bite treatment.

The enrolment of patients with confirmed envenomation is inherently reliant on the need for admission. This excludes patients who sustain “dry bites” from data collection and risk assessment in the overall prevalence of exposure. The small sample size is a reflection of the number of patients admitted for this problem, longitudinal assessment over time will provide a greater sample size as a follow-up. The use of one center for data collection excludes other, larger hospitals; however, the frequency of snakebites at other institutions is notably fewer and would affect overall collection minimally.

## Conclusions

This study took into consideration the patients who had confirmed sustained envenomation with an indication for the hospital admission. This represents a subset of the total number of snake bites which would also include "dry bites" that do not sustain envenomation and those who do not present to the hospital. The data is pertinent to the communities from which the patients are presented, in that large farming communities are serviced by the hospital in question. The data reflects that there are many pre-hospital measures that are taken by patients which have no evidence to support them. They continue to be practiced because of the traditions of rural treatments and a lack of education. This study, therefore, provides evidence that there is an excellent opportunity to begin intensive community education regarding the early and appropriate management of snake bites. It is imperative that this is seen as an occupational hazard for those who work in farming communities and that education programs are designed to formalize the use of protective equipment. Significantly, despite the existence of WHO guidance on the management of envenomation and local protocols regarding the lack of efficacy of prehospital measures, there is a high prevalence of utilization which is directly problematic in terms of precipitating worsening clinical conditions but also indirectly in terms of delayed presentation to hospital. Education programs should be designed therefore to target prehospital providers and community healthcare workers regarding existing evidence.
